# Radioactivity/Radionuclide (U-232 and Am-241) Removal from Waters by Polyurea-Crosslinked Alginate Aerogels in the Sub-Picomolar Concentration Range

**DOI:** 10.3390/gels9030211

**Published:** 2023-03-10

**Authors:** Ioannis Ioannidis, Ioannis Pashalidis, Grigorios Raptopoulos, Patrina Paraskevopoulou

**Affiliations:** 1 Laboratory of Radioanalytical and Environmental Chemistry, Department of Chemistry, University of Cyprus, P.O. Box 20537, Nicosia Cy-1678, Cyprus; 2 Inorganic Chemistry Laboratory, Department of Chemistry, National and Kapodistrian University of Athens, Panepistimiopolis Zografou, 15771 Athens, Greece

**Keywords:** alginate aerogels, polymer-crosslinked aerogels, polyurea-crosslinked alginate aerogels, radioactive decontamination, Am-241 tracers, U-232 tracers, environmental water decontamination

## Abstract

The removal of radionuclide/radioactivity from laboratory and environmental water samples under ambient conditions was investigated via batch-type experiments using polyurea-crosslinked calcium alginate (X-alginate) aerogels. Water samples were contaminated with traces of U-232 and Am-241. The removal efficiency of the material depends strongly on the solution pH; it is above 80% for both radionuclides in acidic solutions (pH 4), while it decreases at about 40% for Am-241 and 25% for U-232 in alkaline solutions (pH 9). This is directly associated with the presence of the radionuclide species in each case; the cationic species UO_2_^2+^ and Am^3+^ prevail at pH 4, and the anionic species UO_2_(CO_3_)_3_^4–^ and Am(CO_3_)_2_^−^ prevail at pH 9. Adsorption on X-alginate aerogels is realized by coordination of cationic species on carboxylate groups (replacing Ca^2+^) or other functional groups, i.e., –NH and/or –OH. In environmental water samples, i.e., ground water, wastewater and seawater, which are alkaline (pH around 8), the removal efficiency for Am-241 is significantly higher (45–60%) compared to that for U-232 (25–30%). The distribution coefficients (*K*_d_) obtained for the sorption of Am-241 and U-232 by X-alginate aerogels are around 10^5^ L/kg, even in environmental water samples, indicating a strong sorption affinity of the aerogel material for the radionuclides. The latter, along with their stability in aqueous environments, make X-alginate aerogels attractive candidates for the treatment of radioactive contaminated waters. To the best of our knowledge, this is the first study on the removal of americium from waters using aerogels and the first investigation of adsorption efficiency of an aerogel material at the sub-picomolar concentration range.

## 1. Introduction

Clean water is essential to health and living organisms are inseparable from water. Therefore, access to safe drinking water is a fundamental human right and a component of effective policy for health protection [1]. The occurrence of natural and artificial radionuclides in drinking water at increased levels can pose a threat to the health of living organisms. The presence of radionuclides in drinking water has attracted growing attention because the quality of the drinking water is related to the health of the citizens [2]. The harmfulness of a radionuclide depends on different parameters including radioactivity levels and the type of radiation emitted. To protect human health and the environment, remediation actions are needed to remove radionuclides from contaminated waters prior to release into environmental receivers and their use for irrigation and drinking purposes [1]. Adsorption-based technologies are of particular interest due to their low cost and easy application, as well as the large spectrum of materials available as adsorbents [3,4].

Among the materials investigated as effective adsorbents are aerogels. Aerogels have been defined as solid colloidal or polymeric networks expanded throughout their entire volume by a gas [5,6]. They are prepared via sol–gel processes, and there are practically no limitations to their chemical composition [7]. They are nanostructured materials with high surface area and high porosity that consist mostly of empty space (>80% *v*/*v*), and therefore they have low bulk densities [8]. Because of these properties, and the fact that their pores cover a large range of micropores, mesopores and macropores, they have been used as adsorbents and in some cases, they have proved to be very efficient.

Indeed, aerogels have shown extraordinary adsorption capacity for various contaminants, including (radio)toxic metal ions (e.g., uranium, thorium, strontium). For uranium sorption, several types of aerogels have been tested in different environments, ranging from laboratory solutions to natural waters (e.g., seawater), or waste waters. These materials include organic, inorganic oxide, chalcogen, carbon and composite aerogels. A detailed presentation of their sorption capacity and the critical parameters that affect their performance is presented in recent publications [9,10]. Among them, hydroxyapatite [11,12], polyurea-crosslinked calcium alginate (X-alginate) [9] and rGO/ZIF-67 (rGO: reduced graphene oxide; ZIF-67: cobalt-based zeolitic imidazole framework) [13] aerogels have sorption capacities close to or above 2000 g/kg (i.e., 2088, 2023 and 1888 g/kg, respectively).

Regarding the sorption of other radionuclides by aerogels, there are only a few references in the literature. For thorium sorption, graphene nanoribbons [14], X-alginate [15] and functionalized graphene oxide composite [16,17] aerogels have been tested with sorption capacities ranging from 38 to 458 g/kg). In addition to the above, the following aerogel adsorbents have also been identified: chalcogen-based (PtGeS, SnS, CoBiMoS, CoNiMoS, CoCrMoS) aerogels for the removal of long-lived radionuclides (e.g., technetium-99, uranium-238, and iodine-129) [18], Prussian blue/cellulose aerogel for cesium capture [19], iron-doped carbon aerogel for strontium capture [20], and X-alginate aerogels for europium capture [15].

Based on the above, it is obvious that the investigation and development of aerogel-based adsorbents for the effective removal of harmful radionuclides from contaminated waters could be very attractive in the treatment of highly contaminated waters (e.g., after a nuclear accident) prior to their discharge to environmental receivers (e.g., rivers or the sea). It is also interesting though to study the removal efficiency of aerogels at ultra-trace levels of radionuclides, which is the most common situation.

In this study, the removal of sub-picomolar concentrations of U-232 (0.1 picomolar) and Am-241 (1 picomolar) from aqueous environments using X-alginate aerogels is being investigated. These radionuclides have been chosen because both are alpha emitters, which have the highest radiological impact after ingestion or inhalation [21], and because they are present in aerobic water environments in the hexavalent and trivalent oxidation state, respectively, which are very common oxidation states for actinides in aqueous solutions [22]. On the other hand, uranium and americium isotopes can been found in the environment at increased levels due to human activities related to the use of nuclear energy and other every days applications (e.g., lightening rod, smoke detectors) [23].

X-alginate aerogels is a new class of biopolymer-based aerogels [24,25,26,27,28] that have been prepared following the polymer-crosslinked (X-aerogel) technology [29,30,31,32,33,34], i.e., via reaction of aliphatic or aromatic triisocyanates with pre-formed M-alginate gels (M refers to divalent or trivalent metal cation), and more specifically, via the reaction of the isocyanate groups with the hydroxy groups of the M-alginate framework and with water that has been retained via hydrogen bonding on the surface of the M-alginate framework. The reaction forms a nano-thin layer of polyurea that covers the M-alginate skeleton without altering the primary M-alginate structure. X-alginate aerogels are mechanically strong materials and extremely stable in various aqueous environments, including seawater [9,35]. They are also environmentally benign, as they consist of calcium alginate, a biopolymer, and polyurea, both of which are non-toxic materials [36,37,38].

**Scheme 1:**
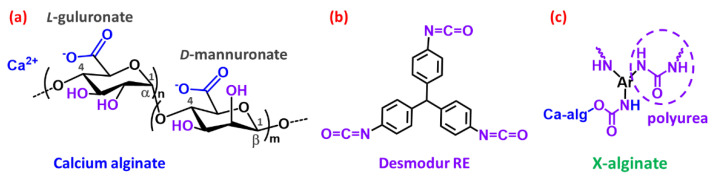
The structures of calcium alginate (**a**), triphenylmethane-4,4′,4″-triisocyanate (TIPM; Desmodur RE) (**b**), and the corresponding X-alginate aerogels (**c**). Calcium alginate is a block copolymer of (1 → 4)-linked *D*-mannuronate and (1 → 4)-linked *L*-guluronate (G).

Therefore, in this study, X-alginate aerogels prepared from the reaction of calcium alginate gels with the aromatic triisocyanate Desmodur RE (Scheme 1) are being investigated towards the removal of very low (sub-picomolar) concentrations of U-232 and Am-241 from aqueous environments. The aqueous phases investigated in this study were laboratory water solutions at three different pH regions (4, 7, 9) and environmental water samples (e.g., seawater, ground water, and treated wastewater). The binding affinity of the studied radionuclides by X-alginate aerogels has been described using the distribution coefficient (*K*_d_). *K*_d_ is defined as the ratio of the radionuclide amount/activity adsorbed per kg of the solid to the radionuclide amount/activity in a liter of the aqueous phase, assuming radionuclide exchange and equilibrium between the two phases. It is a key parameter that describes the aqueous–solid phase distribution and it is widely used in investigations related to radionuclide migration in the geosphere and environmental impact assessments [39]. Moreover, *K*_d_ is of fundamental importance in the development and application of adsorption-based water treatment technologies. To the best of our knowledge, there are no studies on the removal of americium from waters and no investigations at the sub-picomolar concentration range using aerogels as adsorbents.

## 2. Results and Discussion

### 2.1. Effect of Contact Time

**Figure 1:**
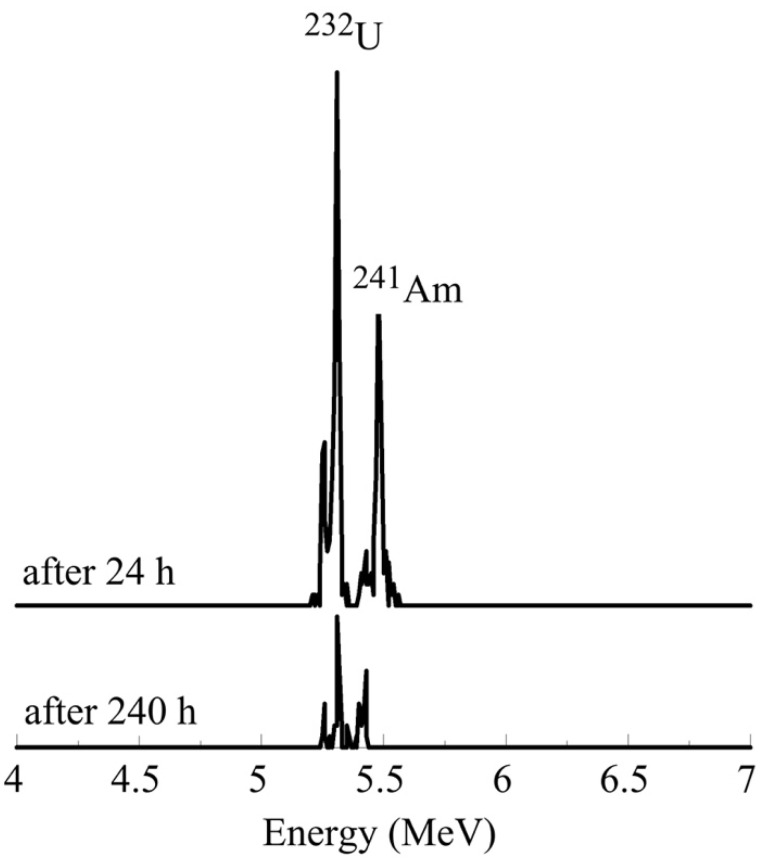
Alpha-spectra of U-232 and Am-241 in solution obtained after adsorption by X-alginate aerogels at two different contact times. Experimental conditions: initial radionuclide concentrations ([U-232] = [Am(241)] = 25 Bq/L), adsorbent dosage 0.26 g/L, pH 4, 296 K and agitation rate 45 rpm.

**Figure 2:**
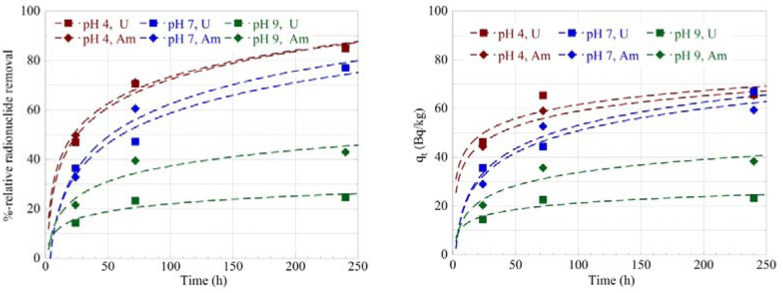
Relative radionuclide (U-232 and Am-241) removal (%; left) and radioactivity removed per kg of aerogel (*q*_t_; right) from laboratory solutions as a function of time and pH, using X-alginate aerogels at 296 K and constant initial radionuclide concentrations ([U-232] = [Am(241)] = 25 Bq/L). Experimental conditions: max contact time 10 days, adsorbent dosage 0.26 g/L, agitation rate 45 rpm.

Τhe relative removal of the U-232 and Am-241 radionuclides from laboratory solutions at three different pH regions has been studied as a function of pH and time and the activity of the remaining U-232 in solution was determined by alpha-spectroscopy (Figure 1) and the corresponding data are graphically summarized in Figure 2. Alpha-spectroscopy was employed for the analysis of the U-232 and Am-241 radioisotopes, because these isotopes are alpha-emitting radionuclides with relatively short half-lives (*t*_1/2_ = 69 and 470 years, respectively). The short half-lives and the very low background noise of alpha-spectroscopy allow detection of the radionuclides in the picomolar concentration range. In addition, the resolution of the semiconductor alpha-detectors enables analysis of mixtures of radionuclides, as those used in the test solutions, and provides a direct comparison of the affinity of the adsorbents for the different radionuclides.

Figure 2 presents the sorption capacity of X-alginate aerogels for the two radionuclides as a function of time and pH, in terms of the relative radionuclide removal (%) and the radioactivity removal per kg of aerogel (*q*_t_ in Bq/kg). According to these data, the equilibrium in the studied systems is reached within 10 days. In our previous studies, which had been performed using X-alginate aerogels for water decontamination from U(VI), Eu(III) and Th(IV) in the mmolar range [9,15], the adsorption equilibrium was reached almost immediately. The slow kinetics observed in the present study is attributed to the diffusion limited adsorption process due to the extremely low radionuclide concentration (in the sub-picomolar range). Similarly, slow adsorption kinetics have been observed when studying radionuclide sorption by microplastics [40,41,42] and biochar materials [43] in a similar concentration range.

**Figure 3:**
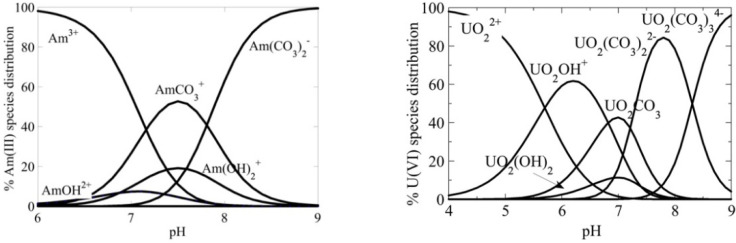
Relative species distribution (%) diagrams for Am(III) and U(VI) as a function of pH in aqueous solutions under ambient conditions.

The removal efficiency for the two radionuclides is almost similar at pH 4 and pH 7 (~80% or 60–65 Bq/kg for both radionuclides) and differs significantly at pH 9 (~25% or 25 Bq/kg for U-232 and ~40% or 40 Bq/kg for Am-241). The removal efficiency declines with pH, indicating its impact on the adsorption process, because pH governs both surface charge of the adsorbent and the radionuclide speciation in solution. Our previous studies [9,15] have shown that adsorption on X-alginate aerogels is realized by coordination of cationic species on carboxylate groups (replacing Ca^2+^) or other functional groups, i.e., –NH and/or –OH. The species of U(VI) and Am(III) that dominate under acidic or alkaline pH conditions are graphically presented in Figure 3, which has been prepared using literature data for U(VI) [44,45] and Am(III) species [46,47,48]. At pH 4, U(VI) and Am(III) exist predominantly in the form of the UO_2_^2+^ and Am^3+^ cations. At pH 7, UO_2_(OH)^+^, UO_2_(OH)_2_ and UO_2_(CO)_3_, and Am^3+^, Am(OH)_2_^+^ and Am(CO_3_)^+^ are the dominating species in solution for U(VI) and Am(III), respectively. However, at pH 9 the predominant species for U(VI) is UO_2_(CO_3_)_3_^4–^ and for Am(III) is Am(CO_3_)_2_^−^. The formation of the negatively charged species stabilizes the radionuclides in solution, resulting in lower removal efficiencies at pH 9.

**Figure 4:**
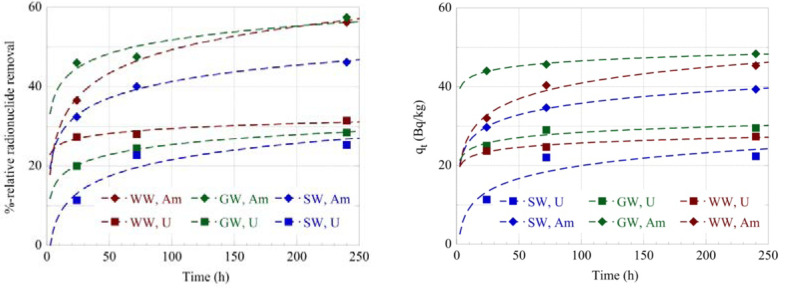
Relative radionuclide (U-232 and Am-241) removal (%; left) and radioactivity removed per kg of aerogel (*q*_t_; right) from environmental waters (seawater (SW), ground water (GW) and wastewater (WW)) as a function of time and pH, using X-alginate aerogels at 296 K and constant initial radionuclide concentrations ([U-232] = [Am(241)] = 25 Bq/L). Experimental conditions: max contact time 10 days, adsorbent dosage 0.26 g/L, agitation rate 45 rpm.

The relative radionuclide removal (%) and the radioactivity removal per kg of aerogel (*q*_t_ in Bq/kg) from environmental waters, e.g., seawater (SW), ground water (GW) and wastewater (WW), are presented in Figure 4. The pH of all these waters is in the alkaline region (7.8–8.3; Table 1). The removal efficiency (25–30% or 20–30 Bq/kg for U-232 and 46–58% or 38–48 Bq/kg for Am-241) in all cases is lower than that determined in acidic and neutral laboratory solutions (Figure 2), and close or even slightly higher (for Am-241) than that at pH 9. This is an indication that even in multicomponent and complex water systems the solution pH and following radionuclide speciation governs the radionuclide removal.

### 2.2. Constants for the Distribution of Am and U between Aqueous and Aerogel Phase

**Figure 5:**
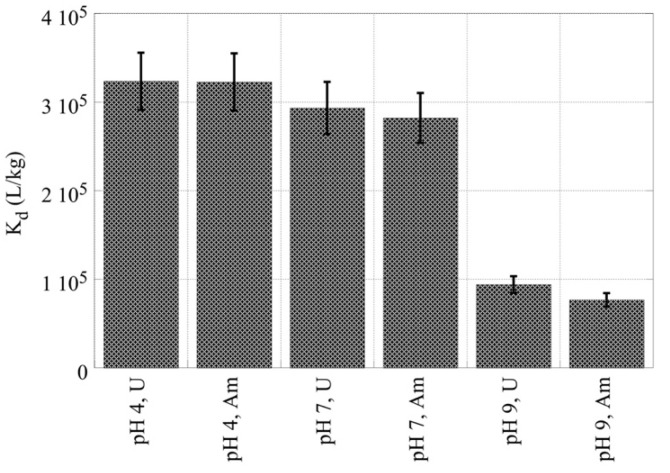
*K*_d_ values for the distribution of U-232 and Am-241 between aqueous lab solutions and X-alginate aerogels as a function of pH at 296 K and constant initial radionuclide concentrations ([U-232] = [Am(241)] = 25 Bq/L). Experimental conditions: contact time 10 days, adsorbent dosage 0.26 g/L, agitation rate 45 rpm.

The *K*_d_ value is a key parameter in modelling radionuclide mobility and migration in the geosphere and of fundamental importance in the assessment and development of adsorption-based water treatment technologies [39]. The distribution coefficient values (*K*_d_), determined for the adsorption of the two radionuclides (Am-241 and U-232) by X-alginate aerogels as a measure for the adsorption efficiency, are reported in Table 1 and are graphically summarized in Figure 4 and Figure 5. In laboratory waters (Figure 4), the *K*_d_ values are above 3 × 10^5^ L/kg at pH 4, decline with increasing pH, and have values below 1 × 10^5^ L/kg at pH 9. As previously discussed, this can basically be attributed to the formation of negatively charged Am(III) and U(VI) species, which stabilize the radionuclides in solution and result in lower adsorption efficiencies. Based on our previous studies on U(VI) and Eu(III) sorption by the same X-alginate aerogels, which included thermodynamic and spectroscopic investigations [9,15], and bearing in mind that (a) the thermodynamics are similar and independent of the concentration [43], and (b) Eu(III) and Am(III) behave similarly in aqueous solutions [49], we assume that sorption of both radionuclides by X-alginate aerogels is an endothermic, entropy-driven process that results in the formation of inner-sphere complexes.

**Figure 6:**
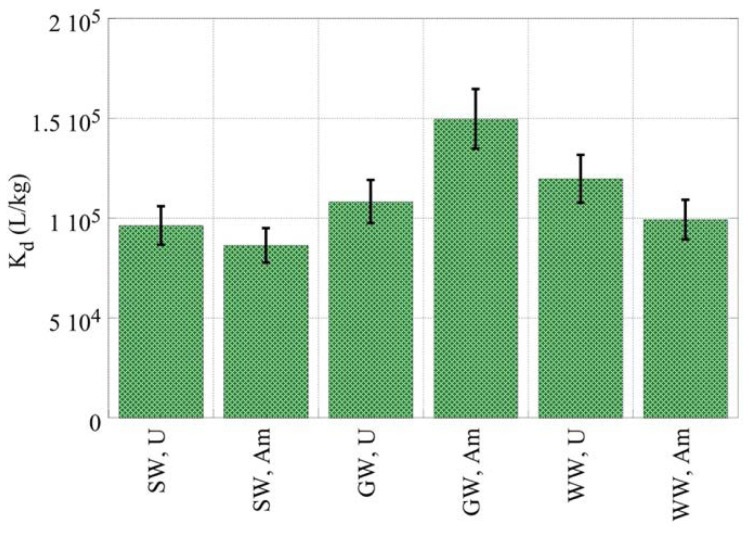
*K*_d_ values for the distribution of U-232 and Am-241 between environmental water solutions (seawater (SW), ground water (GW) and wastewater (WW)) and X-alginate aerogels at 296 K and constant initial radionuclide concentrations ([U-232] = [Am(241)] = 25 Bq/L). Experimental conditions: contact time 10 days, adsorbent dosage 0.26 g/L, agitation rate 45 rpm.

On the other hand, in environmental waters (Figure 6), *K*_d_ values are generally lower than the ones determined in the acidic and neutral laboratory solutions, and closer to the values obtained in the alkaline solutions (pH 9), i.e., around 10^5^ L/kg. This is related to the fact that the specific environmental water samples, i.e., seawater (SW), ground water (GW) and wastewater (WW), have pH values equal to 7.8, 8.1 and 8.3, respectively. These values are in the alkaline pH region, where the anionic radionuclide species (e.g., Am(CO_3_)_2_^−^ and UO_2_(CO_3_)_3_^4–^) are predominant, suggesting that even in multicomponent systems the radionuclide speciation remains the key determining factor for the adsorption of the radionuclides. Another very important factor is the presence of large concentrations of metal ions, which compete with the radionuclides of interest for the coordination sites available on the surface of X-alginate aerogels. This may account for the lower *K*_d_ values calculated for seawater (SW) compared to the other two environmental water samples, as seawater has significantly higher concentrations of metal cations (i.e., Na^+^, K^+^, Ca^2+^, Mg^2+^).

The *K*_d_ values determined for americium in the environmental samples are more than one order of magnitude higher than the best estimate of the distribution coefficient (*K*_d_ = 5 × 10^3^ L/kg) reported for Am(III) in soils [50]. Moreover, the present *K*_d_ values for uranium are significantly higher than the corresponding values determined in soils (including organic soils) and sea sediments (2 × 10^−3^ L/kg < *K*_d_ < 2 × 10^3^ L/kg) [51,52], indicating the superior affinity of X-alginate aerogels for uranium. In addition, these *K*_d_ values are similar to the corresponding values determined for the adsorption of uranium by the best biochar materials (315 L/kg < *K*_d_ < 1.6 × 10^5^ L/kg) [43], and two or three orders of magnitude higher than those evaluated for adsorption by surgical face masks (70 L/kg < *K*_d_ < 80 L/kg) [53] or microplastics (200 L/kg < *K*_d_ < 700 L/kg) [41]. It is obvious that X-alginate aerogels feature high *K*_d_ values, suggesting that these materials could be applied as very effective adsorbents for the treatment of radioactive contaminated waters.

## 3. Conclusions

In this study, the removal of sub-picomolar concentrations of U-232 and Am-241 from aqueous environments using polyurea-crosslinked calcium alginate (X-alginate) aerogels was investigated under ambient conditions via batch-type experiments. The removal efficiency depended strongly on the solution pH, as this affects the speciation of the radionuclides in water. More specifically, the removal efficiency was above 80% for both radionuclides in acidic solutions (pH 4), in which the cationic species UO_2_^2+^ and Am^3+^ prevail, while it decreased at about 40% for Am-241 and 25% for U-232 in alkaline solutions (pH 9), in which the anionic species UO_2_(CO_3_)_3_^4–^ and Am(CO_3_)_2_^−^ predominate. In environmental water samples, i.e., ground water, wastewater and seawater, which are alkaline (pH around 8), the removal efficiency for Am-241 was significantly higher (45–60%) compared to that for U-232 (25–30%). The sorption of both radionuclides by X-alginate aerogels is an endothermic, entropy-driven process. The distribution coefficients (*K*_d_) obtained for the sorption of Am-241 and U-232 by X-alginate aerogels were around 10^5^ L/kg, even in environmental water samples, indicating a strong sorption affinity of the aerogel material for the radionuclides. The latter, along with their stability in aqueous environments, make X-alginate aerogels attractive candidates for the treatment of radioactive contaminated waters. To the best of our knowledge, this is the first study on the removal of americium from waters using aerogels and the first investigation of adsorption efficiency of an aerogel material at the sub-picomolar concentration range.

## 4. Materials and Methods

The standard tracer solutions used were U-232 (National Physical Laboratory, Teddington, UK) and Am-241 (North America Scientific Inc., Los Angeles, CA, USA) with an activity concentration of 4.923 and 12.05 kBq/g, respectively. Tracer standard solutions were used to prepare reference and test solutions, with an initial activity concentration of 25 Bq/L for each radionuclide, which corresponds to about 0.1 pmol/L and 1 pmol/L for U-232 and Am-241 respectively. Sodium alginate PROTANAL LF 240 D (G/M = 0.43–0.54) was used as starting material. Desmodur RE (27% *w*/*w* triphenylmethane-4,4′,4″-triisocyanate (TIPM) solution in ethyl acetate) was generously provided by Covestro AG. MeCN (HPLC grade) was purchased from Fisher Scientific and acetone (P.A., ISO reagent) was purchased from Lach-Ner. Solvents were used as received.

All experiments were performed under ambient conditions (296 ± 2 K) in 20 mL polyethylene screw cap vials. pH measurements were carried out by means of a commercial glass electrode (Sentek), which was calibrated prior and after each measurement using four different buffer solutions (pH 2, 4, 7 and 10, Scharlau).

### 4.1. Preparation and Characterization of X-Alginate Aerogels

X-alginate aerogels were prepared and characterized as described before [26]. The concentration of the initial aqueous solution of sodium alginate was 3% *w*/*w*.

### 4.2. Adsorption Experiments

Adsorption experiments were performed by keeping X-alginate aerogel beads (2.6 mg) in 10 mL of the radionuclide solution containing the uranium and americium isotopes at equal radioactivity concentrations (25 Bq/L). The content of the vials was shaken on a linear shaker (SK-R1807, DLAB) at a constant agitation rate (45 rpm) and temperature (296 ± 2 K). At specified time intervals, an aliquot of 100 μL was withdrawn, the radionuclides were electrodeposited and their concentration in solution was determined by alpha spectrometry (Alpha Analyst Integrated Alpha Spectrometer, Canberra) after calibration of the radiometric system using a calibration source, which was a U-238/234, Pu-239, Am-241 mixed standard on planchet (total activity: 6.6 Bq, Eckert & Ziegler) and a U-232 standard reference solution (1.02 Bq/mL).

The removal efficiency (described using the %-relative amount adsorbed), the time depended adsorption capacity (*q*_t_) and the linear distribution coefficient (*K*_d_) were calculated from Equations (1)–(3), where *V* (L) is the volume of the solution (10 mL), *m* (g) is the mass of X-alginate aerogels (0.0026 g), *C*_o_ (Bq/L) is the initial radionuclide activity concentration (25 Bq/L), *C*_aq,eq_ and *C*_aq,t_ (Bq/L) are the radionuclide activity concentrations at equilibrium and after a certain contact time with X-alginate aerogels, respectively, and *C*_ads_ (Bq/g) is the radioactivity amount of radionuclide adsorbed by X-alginate aerogels at equilibrium.

%-relative removal = 100 × (*C*_o_ − *C*_aq,t_)/*C*_o_ (1)

*q*_t_ = *V* × (*C*_o_ − *C*_aq,t_)/*m* (2)

*K*_d_ = *C*_ads_/*C*_aq,eq_ (3)

The amount of uranium and americium adsorbed by X-alginate aerogels is calculated from the total activity of radionuclides adsorbed minus the relative activity of radionuclides adsorbed by the walls of the vial. The activity of radionuclide adsorbed from the container walls is not negligible and must be taken into account when adsorption experiments are performed using ultra-trace levels. The relative adsorption is calculated as the activity of a radionuclide in the test solution divided by the activity of radionuclide in the reference solution. Experiments were performed in duplicate and mean values have been used for data evaluation and graphical presentations.

### 4.3. Removal of Radionuclides from Environmental Waters

The applicability of X-alginate aerogels beads to remove radionuclides from environmental waters was investigated by contacting seawater (SW), ground water (GW) and wastewater (WW) samples, which were previously contaminated with a defined amount of the radionuclides. Sampling and analysis were carried out according to Standard Methods [54]. Table 2 summarizes the pH values and levels of main components in the studied environmental waters. The uptake experiments were performed as described in Section 4.2, using 0.26 g of X-alginate aerogel beads per liter of solution.

## Figures and Tables

**Table 1:** Distribution coefficient values (*K*_d_) for U-232 and Am-241 adsorption by X-alginate aerogels in laboratory solutions and environmental waters.

	*K*_d_ (L/kg) U-232	*K*_d_ (L/kg) Am-241
**Laboratory solutions**		
pH 4	3.2 × 10^5^	3.2 × 10^5^
pH 7	2.9 × 10^5^	2.8 × 10^5^
pH 9	9.4 × 10^4^	7.7 × 10^4^
**Environmental waters**		
Seawater (SW)	9.6 × 10^4^	8.6 × 10^4^
Ground water (GW)	1.1 × 10^5^	1.5 × 10^5^
Wastewater (WW)	1.2 × 10^5^	9.9 × 10^4^

**Table 2:** pH values and levels of main components in the studied environmental waters (i.e., wastewater (WW), groundwater (GW) and seawater (SW)).

Parameter	WW	GW	SW
pH	8.1	7.8	8.3
K^+^ (mg L^−1^)	29	<3	395
Na^+^ (mg L^−1^)	-	40	10,680
Ca^2+^ (mg L^−1^)	87	38	410
Mg^2+^ (mg L^−1^)	55	70	1280
Fe^3+^ (mg L^−1^)	-	<35	0.003
Cu^2+^ (mg L^−1^)	-	<50	0.09
Cl^−^ (mg L^−1^)	298	54	19,200
HCO_3_^−^ (mg L^−1^)	-	370	140
SO_4_^2–^ (mg L^−1^)	111	95	2680

## Data Availability

The data presented in this study are available on request from the corresponding authors.
